# BRAF V600E in thyroid cancer: navigating prognostic uncertainty and therapeutic opportunity

**DOI:** 10.1530/ETJ-25-0225

**Published:** 2025-12-23

**Authors:** Garcilaso Riesco-Eizaguirre

**Affiliations:** ^1^ Department of Endocrinology and Nutrition, Hospital Universitario de Móstoles, Madrid, Spain; ^2^ Molecular Endocrinology Group, Faculty of Medicine, Universidad Francisco de Vitoria, Madrid, Spain

**Keywords:** thyroid cancer, BRAF V600E, risk stratification, heterogeneity, therapeutic target

## Abstract

This review explores the role of BRAF V600E in thyroid cancer with emphasis on its debated prognostic value, notable molecular heterogeneity, and opportunities as a therapeutic target. BRAF V600E, the most common oncogenic driver in papillary thyroid carcinoma, activates the MAPK pathway and suppresses genes involved in iodine metabolism and differentiation. While linked to adverse features and outcomes such as extrathyroidal extension, lymph node metastasis, recurrence, and mortality, its utility as an independent prognostic marker remains controversial. In solitary intrathyroidal tumors (1–4 cm) and low-risk microcarcinomas, BRAF V600E testing may help refine surgical decisions, though evidence is inconsistent, particularly for tumors <2 cm. The mutation also contributes to radioactive iodine (RAI) refractoriness, but not all BRAF-mutant tumors behave similarly. Transcriptomic and genomic heterogeneity – including differences in thyroid differentiation score, genetic co-alterations and miRNA signatures – modulates treatment response. Targeting BRAF V600E has led to novel therapeutic strategies. Selective BRAF and MEK inhibitors – including vemurafenib, dabrafenib, and selumetinib – have demonstrated efficacy in advanced thyroid cancers. The combination of dabrafenib and trametinib is FDA approved for BRAF V600E-mutant anaplastic thyroid carcinoma based on its significant survival benefits. Moreover, due to its histology-agnostic approval for solid tumors with BRAF V600E mutations, this regimen is now also indicated for papillary and poorly differentiated thyroid cancers. In addition, redifferentiation strategies using MAPK inhibitors to restore RAI avidity have shown promise, particularly in selected patients. These advances highlight the need to contextualize BRAF mutation status within a broader molecular and clinical framework to guide personalized, effective treatment strategies.

## Introduction

BRAF mutations are found in approximately 4–8% of all cancers, with the highest prevalence in melanoma, thyroid cancer, colorectal cancer (CRC), and non-small cell lung cancer (NSCLC) (1, 2). The most common mutation is V600E, which accounts for over half of all BRAF mutations. The BRAF V600E mutation was first identified in thyroid cancer in 2003, showing a prevalence of approximately 45–50% in papillary thyroid carcinoma (PTC), 25–30% in anaplastic thyroid carcinoma (ATC), and absent in follicular thyroid carcinoma and benign thyroid neoplasms (3, 4). The BRAF V600E mutation constitutively activates the MAPK/ERK pathway, leading to uncontrolled cell proliferation, migration, and invasion. Unlike wild-type BRAF, which requires dimerization for activation, BRAF V600E functions as a monomer, resulting in continuous signal transduction without external stimuli, increasing kinase activity by up to 500-fold. BRAF V600E-driven tumors exhibit high MAPK signaling due to a lack of negative feedback regulation from ERK to RAF. This results in enhanced transcriptional gene regulation and reduced expression of genes involved in iodine uptake and metabolism, leading to a lower thyroid differentiation state. By contrast, RAS-driven tumors, the second most prevalent oncogene in thyroid cancer, signal through RAF dimers, which respond to ERK feedback, resulting in lower MAPK signaling and maintaining a more differentiated state (5). While RAS mutations can be present in benign tumors, the presence of BRAF V600E in thyroid nodules is highly specific for malignancy, particularly PTC, and can aid in diagnosing cytologically indeterminate nodules (Bethesda III/IV). However, due to its low prevalence in this setting, its diagnostic utility for avoiding surgery is very limited.

BRAF mutational status is a critical biomarker that informs prognosis and guides personalized treatment strategies across multiple cancers (1). In this review, we analyze BRAF V600E as both a prognostic marker and a therapeutic target in thyroid cancer. First, we examine the evidence supporting the role of BRAF mutation in refining risk stratification, which ultimately influences clinical decision-making. Numerous studies have explored this topic from different perspectives, making it a complex and actively debated area. Second, given that BRAF V600E represents a well-characterized molecular target with available selective inhibitors, we also examine the relevant clinical trials and the emergence of therapeutic strategies that are uniquely tailored to thyroid cancer.

## Prognostic role: BRAF V600E as a marker for risk stratification

### General perspective

In thyroid cancer, there is a notable disconnect between the relatively high recurrence rate (∼20%) and the low mortality rate (<5%), prompting the development of two distinct risk stratification systems. On the one hand, the AJCC/TNM system, designed to predict mortality, underwent significant revisions in its eighth edition, including raising the age cutoff to 55, and excluding microscopic extrathyroidal extension from the T3 definition, and N1 disease no longer upstages a patient to stage III (6). Molecular markers were not incorporated into the AJCC/TNM staging due to insufficient independent prognostic significance, despite their potential for enhancing personalized risk assessments.

On the other hand, the American Thyroid Association (ATA) developed a complementary system targeting recurrence risk. The recent 2025 ATA guidelines for differentiated thyroid cancer (DTC), published shortly after submission of this manuscript, expanded the primary determinants of recurrence risk, integrating histopathologic criteria, AJCC stage, thyroglobulin response, and imaging (7). However, molecular markers, including BRAF V600E, were not routinely recommended for risk stratification or prognosis. Instead, they were considered complementary and investigational for refining prognosis. BRAF V600E is categorized as an ‘intermediate-risk molecular alteration’ – within the broader molecular classification that includes RAS-like (low risk), BRAF-like (intermediate risk), and TERT/TP53/PIK3CA-like (high risk) profiles (7). Still, the role of BRAF V600E in prognostication and treatment decision-making remains a topic of ongoing debate.

Some experts believe that there is enough evidence to support the association of BRAF V600E with aggressive clinicopathologic features, including extrathyroidal extension, lymph node metastases, recurrence, mortality, and resistance to radioactive iodine, suggesting a valuable role in risk stratification and guiding treatment decisions (8). Other experts hold that BRAF V600E alone lacks sufficient specificity and independent prognostic power, as its predictive value often diminishes when adjusted for conventional factors such as tumor size or metastasis; hence, they do not recommend it for use in isolation and suggest it should be considered alongside other genetic alterations for more accurate prognostication (9, 10). This divergence underscores a debate on whether BRAF V600E should serve as a standalone marker or be interpreted within a broader molecular context.

Given the substantial volume of the literature, five meta-analyses published between 2012 and 2016 have evaluated the prognostic relevance of the BRAF V600E mutation in PTC (see Table 1). Collectively, these studies – encompassing cohorts ranging from 2,500 to 25,000 patients approximately – consistently show that BRAF V600E is associated with disease recurrence and adverse pathological features such as extrathyroidal extension and lymph node metastases. However, no consistent association with distant metastases has been observed, and only one meta-analysis reported a link with overall survival (OS), which lacked cause-specific mortality data. While meta-analyses are powerful tools in evidence-based medicine, their interpretation requires caution due to inherent limitations, particularly heterogeneity, publication bias, and variability in study quality. Meta-analysis is a methodological approach rather than a guarantee of high-level evidence; its validity depends on the rigor, design, and consistency of the included primary studies. Therefore, critical appraisal of these findings and emphasis on high-quality primary evidence remain essential (11). In this section, we will examine such primary data in greater detail, including the most recent.

**Table 1:** Comparative summary of BRAF V600E meta-analyses on prognosis.

Meta-analysis	Sample size	Recurrence risk, OR (95%CI)	OS, OR (95% CI)	Distant MTS, OR (95%CI)	Other findings	Limitations
Kim *et al.* (48)	5,655	2.14 (1.67–2.74)	NA	NA	↑ ETE, LNM, TNM stage	Small sample; no OS data
Xing *et al.* (23)	2,470	1.93 (1.61–2.32)	NA	0.95 (0.63–1.44) – NS	↑ ETE, LNM, AJCC stage III/IV	Small sample; heterogeneity; no OS data
Liu *et al*. (66)	14,170	2.50 (1.73–3.59)	NA	Not assessed	↑ ETE, LNM, TNM stage, multifocality	No OS data; recurrence only
Zhang *et al*. (67)	25,241	2.33 (1.71–3.18)	NA	1.23 (0.67–2.27) – NS	↑ ETE, LNM, TNM stage, vascular invasion	High heterogeneity; no OS data
Liu *et al*. (68)	20,764	2.20 (1.57–3.09)	4.61 (2.69–7.90)	1.05 (0.65–1.68) – NS	↑ ETE, LNM, TNM stage	No cause-specific mortality; heterogeneity

NA, not assessed; NS, not significant; MTS, metastasis; OS, overall survival.

### BRAF and mortality

Six major studies have directly addressed the role of the BRAF V600E mutation in relation to mortality in PTC (see Table 2), with findings evolving from early controversy to more nuanced interpretations. Initial studies diverged sharply: while an Italian group identified BRAF V600E as an independent predictor of persistent disease and mortality (13.1% mortality in mutated vs 1.6% in wild-type) (12), a Japanese group found no correlation between the mutation and either high-risk pathological features or disease-free survival, suggesting that BRAF alone may not be a robust prognostic marker (13). However, subsequent large-scale, multicenter efforts led by M Xing’s group at Johns Hopkins Hospital helped clarify these discrepancies. This group reaffirmed an association between BRAF and disease-specific mortality (5.3 vs 1.1%), though this effect was not independent after adjusting for tumor aggressiveness (lymph node metastasis (LNM), extrathyroidal invasion, and distant metastasis), implying that BRAF’s role may be mediated through downstream pathology (Table 2) (14).

**Table 2:** Comparative summary of studies addressing BRAF association with mortality.

Study	Sample size, *n*	Centers, *n*	Median FUT	Main clinical outcomes	Key limitations
Elisei *et al.* (12)	102	1	15 years	BRAF V600E significantly associated with persistent disease (21.1%) and mortality (13.1%); independent predictor of poor outcome	Small sample size; potential selection bias; effect of treatment standardization not clear
Yasuhiro *et al.* (13)	631	1	83 months	No correlation between BRAF V600E and high-risk features or disease-free survival; similar DFS and distant metastasis-free survival in both groups	BRAF not associated with outcomes possibly due to surgical strategy; results may reflect Japanese treatment patterns, limiting generalizability
Xing *et al.* (14)	1,849	13	36 months	BRAF V600E is associated with increased mortality (5.3 vs 1.1%, HR 2.66), but not independent after adjusting for aggressive tumor features	Association weakened after adjusting for aggressive features
Shen *et al.* (15)	2,638	11	37 months	Mortality increases linearly with age only in BRAF-mutated patients (0.34% < 45 years vs 13.33% > 75 years). No age effect in BRAF wild-type	Mortality low in young patients limiting stratified analysis; limited molecular profiling beyond BRAF
Wang *et al.* (16)	2,638	11	37 months	Male sex increases mortality only in BRAF-mutated patients (6.6 vs 2.9%, HR 2.43); no sex difference in wild-type	Confounding by unmeasured variables
Tao *et al.* (17)	2,638	11	37 months	Lymph node metastasis increases mortality only in BRAF-mutated patients (7.7 vs 1.4%, HR 27.39); no effect in wild-type	Omission of key confounders such as distant metastasis; unclear if LNM directly increases mortality; inconsistency with the recurrence-focused literature

FUT, follow-up time.

Later studies led by the same group integrated clinical modifiers – age, sex, and LNM – into the BRAF prognostic framework, offering a more precision-medicine-oriented perspective (Table 2). Shen *et al.* demonstrated that age-driven mortality was specific to BRAF-mutant cases, challenging the universal staging reliance on age (15). Similarly, Wang *et al.* found that male sex conferred higher mortality only in BRAF-mutated PTC (16), and Tao *et al.* showed that LNM predicted mortality only in BRAF-mutant tumors, with a staggering HR of 27.39 (17). These studies collectively argue that traditional risk factors (age, sex, and LNM) acquire lethal significance only in the molecular context of BRAF V600E. Still, controversy persists: the Tao study has drawn criticism for not adjusting for distant metastases and tumor stage, and for attributing dramatic mortality risk to LNM, a factor typically more associated with recurrence than death (18). Altogether, these findings underscore the importance of molecular stratification in thyroid cancer prognosis, while also highlighting the limitations of retrospective design and the need for prospective validation to resolve ongoing debates.

Shortly after submission of this manuscript, a recent large multicenter retrospective cohort study integrating genetic and clinical data from 4,746 PTC patients examined whether incorporating BRAF V600E and TERT promoter mutations could improve the prognostic accuracy of the AJCC/TNM staging system for thyroid cancer–specific mortality (19). The study, led by M Xing’s group, showed that BRAF V600E alone did not significantly predict mortality; however, integrating BRAF and TERT genotyping into the AJCC/TNM system substantially enhanced mortality risk stratification, particularly in stages I–III, where the coexistence of both mutations effectively upgraded stage classifications (I → II, II → III, and III → IV). Taken together, these findings underscore that the clinical impact of BRAF V600E depends strongly on the presence of other genetic alterations.

### BRAF and recurrence

The largest and most robust analysis to date on the prognostic value of the BRAF V600E mutation for recurrence in PTC was also conducted by the research group at Johns Hopkins Hospital led by M Xing. It was a retrospective multicenter study involving over 2,000 patients from 16 institutions worldwide (20). It offered robust insights into the mutation’s role in recurrence prediction, overcoming limitations of prior smaller studies. Mutation-positive patients showed a significantly higher recurrence rate (20.9%) compared to mutation-negative patients (11.6%). Even after multivariable adjustments for factors such as age, tumor size, and LNM, the mutation retained significance (HR = 1.82, *P* < 0.05). This association holds across clinicopathologic risk factors, including low-risk groups (e.g., stage I or II, micro-PTC) and subtypes such as conventional and follicular-variant PTC. Along with the previously mentioned meta-analyses, these results support the mutation as an independent prognostic marker for improving risk stratification and treatment planning in PTC patients and have been confirmed by other studies (8). However, some studies are not consistent with these findings, and some authors claim that, while the mutation correlates with higher recurrence rates, its effect size is quite modest, particularly when compared to other clinicopathologic markers, suggesting that testing should remain limited until further prospective studies confirm its clinical value (9).

In a recent prospective study in patients with low-risk thyroid cancer who had undergone total thyroidectomy for tumors measuring 2 cm or less, the ESTIMABL2 trial, a nested molecular analysis was performed on 90 tumor samples from a total of 96 patients (cases with events and matched controls without events) (21). Of these, 50 tumors (55.6%) harbored the BRAF V600E mutation. The mutation was present in 61.5% of patients who experienced an event (recurrence or abnormal thyroglobulin levels) and in 53.1% of patients who remained event-free, indicating no meaningful difference in recurrence risk based on BRAF status. As a result, the study concluded that in a well-defined population of low-risk DTC patients, the presence of BRAF V600E should not be used as an indication for postoperative radioiodine treatment, as it did not correlate with poorer outcomes or increased recurrence risk during the 3-year follow-up period.

In another recent study, the role of the BRAF V600E mutation in the risk assessment and dynamic risk stratification (DRS) of PTC was systematically evaluated across a large, real-world, multicenter Spanish cohort (*n* = 685) (22). The BRAF V600E mutation was associated with extrathyroidal extension, lymph node metastases, and a higher rate of biochemical incomplete response to therapy (HR = 2.43; 95% CI: 1.21–5.23; *P* = 0.016). Although BRAF mutation increased the risk of persistent/recurrent disease in univariate and age/sex-adjusted models, its significance disappeared after adjusting for other aggressive clinicopathologic factors (OR = 1.57; 95% CI: 0.95–2.62; *P* = 0.078). BRAF status did not correlate with structural incomplete response or distant metastases, and its incorporation into DRS did not improve the prediction of recurrence beyond DRS alone. Thus, while BRAF is linked to intermediate-risk features and local aggressiveness, it does not provide additional prognostic value when integrated into response-to-therapy-based risk models.

Finally, it is important to consider subsequent evidence from Mingzhao Xing’s group showing that the prognostic meaning of *BRAF V600E* depends strongly on the presence of other genetic alterations, particularly *TERT* promoter mutations. While early studies suggested an association between *BRAF V600E* and poorer clinicopathological outcomes, later large-scale analyses incorporating *TERT* promoter mutations (23, 24) demonstrated that the prognostic effect of *BRAF V600E* alone is modest and often loses statistical significance after adjustment for established risk factors. By contrast, the coexistence of *BRAF V600E* and *TERT* promoter mutations identifies a distinct high-risk molecular subset of PTC, in which synergistic activation of oncogenic and telomerase pathways drives substantially higher recurrence rates and poorer outcomes.

Nevertheless, there are two clinical scenarios where BRAF V600E may help to nuance the risk of recurrence and determine treatment decision-making.

#### BRAF V600E in solitary intrathyroidal PTC

The 2015 ATA guidelines recommended lobectomy as an option for solitary intrathyroidal PTC (SI-PTC; lacking LNM, extrathyroidal invasion, and distant metastasis) of tumor size larger than 1.0 and 4.0 cm or less (25). This recommendation has gained increasing popularity over the past decade and remains essentially the same in the 2025 ATA guidelines (7, 26). Two studies have explored the value of BRAF mutation in refining risk stratification for SI-PTC 1–4 cm that can be specifically applied to guide surgical decisions between lobectomy and total thyroidectomy in this clinical setting. In a 2012 study involving 319 patients with T1–T2,N0,M0 PTC, BRAF V600E was present in 33.2% and was associated with adverse histopathological features such as multifocality, aggressive variants, and capsular invasion (27). Notably, disease persistence at 5 years was significantly higher in BRAF-positive patients (16%) compared to BRAF-negative patients (3.3%), making BRAF V600E the only independent predictor of persistent disease. The authors highlighted its strong negative predictive value (NPV) (96.7%) and suggested that BRAF-negative patients could potentially benefit from more conservative treatments.

Building on these findings, a large multinational study in 2017 analyzed 955 patients with SI-PTC across 11 centers in six countries (28). Tumors were classified by size (1–4 cm) and analyzed for recurrence rates and prognostic differences between BRAF V600E mutation-positive and mutation-negative patients. It confirmed a higher recurrence rate in BRAF-mutant tumors (9.5 vs 3.4%), especially in tumors >2 cm (16.5 vs 3.6%) and >3 cm (30 vs 1.9%), with adjusted hazard ratios up to 14.7. Mutation-positive SI-PTC (especially 2–4 cm tumors) had recurrence rates comparable to invasive PTC, favoring total thyroidectomy over lobectomy for these patients. Thus, mutation-negative SI-PTC showed very low recurrence rates, supporting lobectomy for preserving thyroid function and reducing surgical risks. Apart from informing the extent of thyroidectomy, the study suggests tailoring radioiodine ablation and lymph node dissection based on BRAF status. Together, these two studies provide evidence for incorporating BRAF V600E testing in clinical decision-making for low-risk PTC to better tailor the extent of surgery and radioiodine use.

However, this perspective is not universally accepted. As all patients in both studies underwent total thyroidectomy (± radioiodine therapy), it remains unclear whether BRAF-negative patients would have similarly low recurrence risk if managed with less extensive surgery, such as lobectomy. Furthermore, the studies do not provide enough detail on the anatomical sites of recurrence (e.g. central vs lateral neck), limiting the ability to translate findings into decisions such as prophylactic lymph node dissection (29). In addition, some authors caution against overinterpreting BRAF V600E as a standalone marker of poor prognosis, especially in tumors smaller than 2 cm (21). They argue that lobectomy remains appropriate for many such patients, emphasizing that the mutation’s prognostic power is limited unless it coexists with other high-risk features such as TERT promoter mutations (26). Moreover, recent research has broadened the indications for lobectomy, extending its use to patients with contralateral benign nodules and several ‘adverse’ histological features, such as minimal extrathyroidal extension, small lymph node metastases, or multifocality – provided these are of minimal size (26). These ongoing divergences in interpretation underscore the need for further prospective studies to guide molecularly informed treatment decisions.

#### *BRAF V600E in low-risk* papillary thyroid microcarcinoma (PTMC)

The prognostic significance of the BRAF V600E mutation in PTMC has been extensively studied, with early single-center investigations reporting conflicting results. Table 3 summarizes the main studies. Studies from South Korea and Poland in 2013 found high prevalence rates of BRAF V600E (over 69%) but no significant association with adverse features or recurrence, even over long-term follow-up, suggesting a limited prognostic role in indolent, low-risk PTMC (30, 31). Subsequent meta-analyses, however, provided a broader statistical perspective, revealing that BRAF V600E was significantly associated with multifocality, extrathyroidal extension, LNM, and advanced stage, as well as a doubling of recurrence risk (32, 33). These findings indicated a correlation with more aggressive behavior, though this was detectable primarily through pooled data rather than individual studies.

**Table 3:** Summary of studies on BRAF V600E in papillary thyroid microcarcinoma (PTMC).

Study	Patients, *n*	Study design	BRAF V600E prevalence	Main outcome	Clinical implications
Choi *et al.* (31)	101	SC-RCS	71.3%	No significant association with adverse features or recurrence	Limited prognostic value in isolation
Walczyk *et al.* (30)	113	SC-RCS	69%	No recurrence or death; BRAF status not prognostic	BRAF not useful in low-risk PTMC
Li *et al.* (32)	3,437	MA	Varied across studies	Associated with multifocality, ETE, LNM, and advanced stage	Supports BRAF as a risk marker in aggregated data
Chen *et al.* (33)	2,247	MA + PDI	Varied across studies	BRAF linked to 2x recurrence risk (OR: 2.09)	Potential preoperative risk marker for recurrence
Kim *et al.* (34)	743	MC-RCS	32.4%	Higher recurrence in BRAF+ (4.3 vs 1.3%); NPV 98.7%	Supports surveillance in BRAF–; BRAF + may need intervention
Perera *et al.* (35)	111	CCS	61%	BRAF alone not predictive; BRAF + TERT/TP53 linked to aggressive disease	Suggests molecular classifiers outperform BRAF alone

SC-RCS, single-center retrospective cohort study; MC-RCS, multicenter retrospective cohort study; MA, meta-analysis; CCS, genomic and transcriptomic profiling case/control study; MA + PDI, meta-analysis with primary data integration.

A large retrospective 2020 multicenter study including 743 patients offered clinical nuance by showing that BRAF V600E had prognostic significance only in low-risk PTMC, where recurrence was 4.3% in BRAF-positive vs 1.3% in BRAF-negative patients, yielding a hazard ratio of 6.65 and an NPV of 98.7% for wild-type BRAF (34). These findings suggest that conservative active surveillance is a reasonable option for BRAF-negative low-risk PTMC, given the robust NPV and overall excellent prognosis. In contrast, the increased recurrence risk and other well-documented adverse features of BRAF V600E, such as extrathyroidal extension and LNM, make the feasibility of long-term surveillance more uncertain in BRAF-positive cases. However, critics argue that the low absolute recurrence rates do not justify changes in management and raise concerns that routine BRAF testing could lead to overdiagnosis, unnecessary thyroidectomies, and increased healthcare costs without clear survival benefits. Notably, a previous study by the same group found extremely low recurrence rates in PTC <1 cm, and BRAF V600E did not significantly impact outcomes, suggesting that the mutation may have limited prognostic value in microcarcinomas and highlighting a potential inconsistency in the clinical utility of BRAF across patient cohorts (28).

Finally, a 2019 genomic and transcriptomic profiling study of 111 PTMC patients provided further insights. In this case/control study, BRAF mutations were detected in 61% of tumors, with no significant frequency difference between metastatic (pN1b) and non-metastatic (pN0) groups (35). However, co-occurrence of BRAF with TERT or TP53 mutations was exclusive to high-risk pN1b tumors and associated with aggressive histological features and poor outcomes, including anaplastic transformation and early death. Furthermore, a gene expression–based random forest classifier achieved 91% predictive accuracy, suggesting that RNA-based models may better stratify metastatic risk than DNA mutations alone.

#### Concluding remarks

In conclusion, while the BRAF V600E mutation has consistently shown an association with adverse pathological features and increased recurrence risk in PTC, its clinical utility as a standalone prognostic marker remains limited and context dependent. Retrospective and meta-analytic data support its role in refining risk stratification, particularly in tumors 2–4 cm and solitary intrathyroidal cases, where BRAF status may help guide more aggressive initial treatment, such as total thyroidectomy. Yet, prospective studies – especially in low-risk <2 cm or microcarcinoma populations – have not confirmed a consistent impact on outcomes. Furthermore, its prognostic value is significantly enhanced when evaluated alongside other molecular alterations, such as TERT or TP53 mutations. Therefore, although BRAF V600E testing may inform management decisions in selected clinical scenarios (i.e. 2–4 cm SI-PTC), broader molecular profiling and prospective validation are essential for its integration into personalized therapeutic strategies.

### BRAF V600E in predicting radioiodine refractoriness of thyroid cancer

The association between the BRAF V600E mutation and RAI refractoriness was first described in 2005 (36). This mutation suppresses the expression of genes involved in iodine metabolism, impairing the efficacy of RAI therapy. Experimental models have shown that introducing BRAF V600E into normal thyroid cells reduces iodine-processing gene expression, while MAPK pathway inhibition can restore iodine uptake. Despite reduced RAI avidity, some authors advocate RAI treatment in BRAF V600E-positive PTC, as complete loss of uptake is uncommon (8).

It is important to note that rather than an all-or-nothing phenomenon, RAI refractoriness appears to be a spectrum. Some tumors exhibit only partial iodine uptake loss. The TCGA study introduced the thyroid differentiation score (TDS), derived from iodine metabolism gene expression, to stratify PTCs by their differentiation status (5). While RAS-driven tumors tend to be genetically homogeneous and maintain differentiation with a higher TDS, BRAF V600E-mutated PTCs, although globally exhibiting a lower TDS, are highly heterogeneous, exhibiting diverse genetic, epigenetic, and differentiation profiles. TCGA identified four molecular subtypes within BRAF V600E PTCs, characterized by differences in gene expression, DNA methylation, and miRNA profiles. For instance, increased levels of oncogenic miRNAs (e.g. miR-21 and miR-146b) and reduced tumor-suppressive miRNAs (e.g. miR-204) were linked to dedifferentiation and aggressive behavior (5, 37).

Expanding on this heterogeneity, a subsequent study on 227 BRAF-mutant PTCs from the TCGA study classified BRAF-mutant PTCs into two groups based on TDS: BRAF-TDShi (∼17%) and BRAF-TDSlo (∼83%) (38). The TDShi group showed higher differentiation, smaller tumors, fewer lymph node metastases, and significantly better treatment response (94% complete response vs 57% in TDSlo). TDShi tumors also overexpressed miR-204, miR-205, and miR-144, which may regulate the TGFβ-SMAD pathway – previously implicated in thyroid differentiation in BRAF-driven tumors (39, 40). These findings emphasize that not all BRAF-mutant tumors have the same prognosis and that incorporating TDS could improve risk stratification and treatment decisions.

Importantly, RAI responsiveness is not determined by BRAF status alone. A recent retrospective case–control study comparing exceptional responders (ERs; *n* = 8) and non-responders (NRs; *n* = 16) to RAI in metastatic thyroid cancer highlighted additional genomic drivers (41). While ERs harbored alterations that activated MAPK signaling through RAF dimerization (e.g. RAS mutations, class 2 BRAF, and RTK fusions), NRs were enriched for BRAF V600E and additional high-risk alterations. These included chromosome 1q gain – detected exclusively in NRs – loss-of-function mutations in chromatin remodeling genes of the SWI/SNF complex (ARID1A/B), PI3K pathway mutations (PIK3CA and PTEN), and RNA splicing factors (e.g. RBM10). These alterations were associated with low TDS and poorer outcomes. Surprisingly, TERT promoter mutations, although frequently co-occurring with BRAF V600E and associated with increased recurrence risk, were found in both ERs and NRs and did not correlate with RAI refractoriness.

In summary, RAI refractoriness in PTC – particularly BRAF V600E-driven tumors – is a multifactorial and heterogeneous process. Tumor behavior is shaped not only by MAPK pathway activation but also by the degree of differentiation, miRNA expression, and co-occurring genetic and epigenetic alterations. Tools such as the TDS and the identification of additional molecular markers (e.g. 1q gain, PI3K pathway mutations, and SWI/SNF complex alterations) enable a more refined prediction of RAI response. This supports a shift from binary RAI classification toward a spectrum-based, personalized model that better informs prognosis and therapeutic strategies.

## Therapeutic role: BRAF V600E as a therapeutic target

The unraveling of the therapeutic role of BRAF in advanced thyroid cancer followed a successful process of translational research. As in other cancer types in which BRAF was shown to be a prominent oncogene, it was soon clear in preclinical studies that BRAF V600E signaling through MEK-ERK was essential for driving thyroid oncogenesis; inhibiting such signaling in genetically engineered mouse models expressing BRAF V600E blocked tumor transformation and paved the way for the development of specific inhibitors as a new strategy for the treatment of thyroid cancer. Three of them, selumetinib, vemurafenib, and dabrafenib, have been clinically tested in BRAF-mutant thyroid cancer patients.

### Selumetinib

Selumetinib (AZD6244) is a selective MEK1/2 inhibitor, targeting the mitogen-activated protein kinase (MAPK) pathway, specifically blocking MEK1 and MEK2 kinases. By inhibiting MEK, it prevents the activation of ERK1/2, disrupting downstream signaling involved in cell proliferation and survival, particularly in cancers driven by RAS and BRAF mutations.

The therapeutic potential of selumetinib in RAI-refractory thyroid cancer has been explored through two main strategies: as a direct antitumor agent, and as a redifferentiation agent to enhance the efficacy of RAI therapy (Table 4). The rationale for the latter approach was established in a preclinical study by Chakravarty *et al.*, which demonstrated that thyroid cancers driven by BRAF V600E mutations in a mouse model exhibit profound suppression of sodium-iodide symporter expression, rendering them resistant to RAI. However, pharmacological inhibition of the MAPK pathway with MEK or BRAF inhibitors successfully restored iodine uptake, leading to tumor regression after RAI therapy. These findings provided compelling evidence that MEK inhibition could potentially resensitize BRAF-mutant tumors to RAI, thus setting the stage for clinical trials (42).

**Table 4:** Clinical trials of selumetinib in thyroid cancer.

Study	Phase	Setting/population	Strategy	Treatment duration	Key clinical outcomes	BRAF-mutant subgroup findings
Hayes *et al.* (43)	II	39 patients (32 evaluable) with IR-PTC	Direct antitumor effect	Continuous (until progression/toxicity)	PR: 3%; SD: 54%; median PFS: 32 weeks	Longer PFS in BRAF-mutant tumors (33 vs 11 weeks)-NSS
Ho *et al*. (44)	Pilot	20 patients with metastatic IR-DTC	IU modulation for RAI	4 weeks	Increased IU in 12/20 (60%); 8/20 treated with RAI → 5 PR, 3 SD; avg. Tg reduction: 89%	4/9 (44%) BRAF-mutant responded; 5/5 (100%) NRAS-mutant responded
Ho *et al.* (45) (ASTRA)	III	233 patients with high-risk resected DTC (adjuvant setting)	IU modulation for RAI	5 weeks	CR at 18 months: 40% (selumetinib) vs 38% (placebo); OR = 1.07, *P* = 0.82; grade ≥3 AEs: 16%	IU increased in 1/11 (9%) BRAF-mutant patients; no CR benefit
Wadsley *et al.* (46) (SEL-I-METRY)	II	Planned: 60 pts	IU modulation for RAI	4 weeks	IU increase in 9/28 (32.1%)	1/5 (20%) BRAF-mutant achieved sufficient IU
		Completed: 28 evaluable RAI-refractory DTC			12-month PFS (RAI-treated): 64.8%; radiologic response: 22%; grade 3 AEs: 46%	
Taprogge *et al.* (47)	–	14 patients from SEL-I-METRY trial	Dosimetry assessment	–	Absorbed doses <1 to 1,170 Gy; correlation (predicted vs delivered): *r* = 0.93, *P* < 0.001	Not separately analyzed; supports need for individualized planning

PR, partial response; SD, stable disease; IR, iodine refractory; IU, iodine uptake; NSS, not statistically significant.

The first clinical trial evaluating selumetinib as a direct antitumor agent was a phase II study involving 39 patients with iodine-refractory PTC. The trial demonstrated modest clinical activity, with a 3% partial response rate, 54% stable disease, and a median progression-free survival (PFS) of 32 weeks. A trend toward improved PFS was noted in BRAF-mutant tumors (33 vs 11 weeks), though not statistically significant (43). This strategy required continuous treatment, and although toxicity was manageable, the modest efficacy indicated that selumetinib alone may not be sufficient to control disease progression.

Recognizing the limitations of selumetinib alone, subsequent studies shifted focus toward its role in restoring iodine uptake to enable effective RAI therapy, yielding more promising results. The pivotal pilot study by the group of J Fagin at MSKC tested whether selumetinib could reinduce iodine avidity in 20 patients with iodine-refractory metastatic thyroid cancer. Remarkably, 12 of 20 patients (60%) showed increased iodine uptake after treatment, and 8 patients (40%) reached the dosimetry threshold for RAI administration. Of these 8 treated with RAI, 5 achieved partial responses, and 3 had stable disease, with an average thyroglobulin reduction of 89%. Interestingly, all 5 patients with NRAS mutations responded (100%), while only 4 of 9 BRAF-mutant patients (44%) showed a clinically meaningful response. This differential effect suggested that BRAF-mutant tumors may require additional or alternative pathway inhibition to fully restore iodine uptake, a concept that would influence later clinical trial designs (44).

The most extensive investigation of this approach was conducted in the ASTRA trial, aimed at testing selumetinib in an adjuvant setting. It enrolled 233 patients with high-risk DTC who were candidates for adjuvant RAI therapy after initial surgery (45). Patients were randomized to receive selumetinib or placebo for 5 weeks before a standard empirical dose of RAI, and the primary endpoint was complete response (CR) at 18 months, defined by structural and biochemical remission. The trial found no significant difference in CR rates between groups (40% with selumetinib vs 38% with placebo). In the subgroup of patients with BRAF-mutant tumors, selumetinib did not improve outcomes, and iodine uptake increased in only 1 of 11 patients, confirming a low resensitization rate in this genotype. These results suggest that the adjuvant use of selumetinib in unselected or BRAF-mutant populations may be less effective, and that the redifferentiation strategy may be more suited to treating metastatic disease.

The SEL-I-METRY phase II trial, a UK multicenter study, further explored the use of selumetinib as a resensitizing agent in patients with RAI-refractory DTC metastatic disease (46). The trial initially planned to recruit 60 patients, with the expectation that 38 (60%) of them would show increased iodine uptake following a 4-week course of selumetinib 75 mg twice a day, forming the so-called iodine-uptake cohort eligible for subsequent RAI therapy. However, the study was closed prematurely due to low patient accrual and a lower-than-expected iodine uptake response, with only 28 evaluable patients ultimately enrolled. Among these, nine patients (32.1%) demonstrated sufficient iodine uptake to proceed to RAI, substantially less than the anticipated 60%. Among patients with known BRAF mutations (*n* = 5), only one (20%) achieved sufficient uptake, again reflecting a lower responsiveness in this subgroup (46). Despite these limitations, patients who received RAI experienced a 12-month PFS of 64.8% and 22% achieved a radiologic response. However, toxicity was notable, with 46% experiencing grade 3 adverse events, and a high frequency of dose reductions and early discontinuation. Complementing these findings, a dosimetry analysis in 14 patients from the same trial showed a broad range of absorbed doses to metastases (<1 to 1,170 Gy) and a strong correlation (*r* = 0.93, *P* < 0.001) between predicted and delivered doses, supporting the use of pretherapeutic dosimetry to optimize patient selection and therapeutic outcomes (47).

Collectively, these studies show that while selumetinib as monotherapy (direct antitumor agent) offers limited benefit, its use as a redifferentiation agent, particularly when integrated with molecular profiling and individualized dosimetry provides a more promising approach. Yet, variability in response, especially in BRAF-mutant tumors, and the toxicity observed in broader populations highlight the need for refined patient selection and optimized combination strategies to fully harness the potential of this therapy.

### Vemurafenib

Vemurafenib is a selective BRAF inhibitor that specifically targets the BRAF V600E mutation. By inhibiting mutated BRAF, it prevents downstream MEK-ERK activation, reducing tumor cell proliferation and survival in cancers dependent on aberrant BRAF signaling, such as melanoma. Table 5 summarizes the main clinical trials in thyroid cancer.

**Table 5:** Clinical trials summary for vemurafenib.

Study	Phase	Population (patients, *n*)	Primary endpoint	Key clinical outcomes
Kim *et al.* (48)	I	Metastatic BRAF V600E-mutant PTC (*n* = 3)	ORR	1 PR (31%), 2 SD; PFS: 11.4–13.2 months
Hyman *et al.* (49)	II*	BRAF V600E-mutant ATC (*n* = 7)	ORR	1 PR, 4 SD, 2 PD; median duration of response: 6.7 months
Brose *et al.* (50)	II	BRAF V600E-positive, RAI-refractory PTC (*n* = 51)	ORR	ORR: 38.5%; PFS: 18.8 months; grade 3–4 toxicity: 65%, SCC: 27%
Cabanillas *et al.* (51)	II	BRAF V600E-mutant ATC (vemurafenib + cobimetinib + atezolizumab) (*n* = 42)	OS improvement vs historical controls	Median OS: 43 months; PFS: 13.9 months; achieved primary endpoint
Dunn *et al.* (52)	PS	BRAF V600E-mutant, RAI-refractory PTC (*n* = 12)	Increase in RAI uptake	4/10 patients increased RAI uptake; 4 treated with I-131; tumor regression at 6 months
Tchekmedyian *et al.* (54)	PS	BRAF V600E-mutant, RAI-refractory PTC (vemurafenib + anti-HER3) (*n* = 7)	Increase in RAI uptake	5/7 patients increased RAI uptake; 4 treated with I-131; 2 PR, 2 SD at 6 months

* Basket study.

PS, pilot study; ORR, objective response rate; OS, overall survival.

The first clinical evidence of vemurafenib’s efficacy in thyroid cancer was reported by Kim *et al.*, who conducted a phase I dose-escalation study in three patients with metastatic BRAF V600E-mutant PTC (48). One patient achieved a confirmed partial response with a 31% reduction in pulmonary lesions, while two others exhibited stable disease. Time to progression ranged from 11.4 to 13.2 months, indicating potential clinical benefit but also highlighting the limitations of single-agent vemurafenib. A subsequent basket trial included seven patients with ATC, among whom one achieved a partial response, four had stable disease, and two experienced disease progression. The median duration of response in this ATC cohort was 6.7 months (49). A larger phase II trial in 51 patients with RAI-refractory thyroid cancer reported an objective response rate (ORR) of 38.5% in patients without prior multikinase inhibitor (MKI) therapy, with a median PFS of 18.8 months. However, the study also noted significant toxicities, with 65% of patients experiencing grade 3–4 adverse events, including squamous cell carcinoma (27%) and lymphopenia (8%) (50). More recently, combination therapy incorporating vemurafenib with the MEK inhibitor cobimetinib and the immune checkpoint inhibitor atezolizumab in 42 patients with ATC demonstrated a substantial survival benefit, with a median OS of 43 months and a PFS of 13.9 months, greatly exceeding the historical OS of 5 months (51).

As with selumetinib, a second therapeutic strategy sought to enhance RAI uptake in refractory thyroid cancers by leveraging vemurafenib-induced redifferentiation seen in genetically engineered mouse models with conditional BRAF V600E activation (42). A pilot study of 12 patients with BRAF V600E-mutant, RAI-refractory thyroid cancer demonstrated that 4 out of 10 evaluable patients exhibited increased iodine uptake on iodine-124 PET scans, leading to therapeutic I-131 administration and tumor regressions at 6 months (52). However, response variability highlighted the need for additional strategies to overcome resistance mechanisms. Further research identified a feedback loop in thyroid cancer cells, where RAF and MEK inhibition led to HER3 upregulation, ultimately reactivating the MAPK pathway and limiting the sustained effects of BRAF inhibition (53). This finding led to a pilot study combining vemurafenib with an anti-HER3 monoclonal antibody in seven patients with BRAF V600E-mutant, RAI-refractory thyroid cancer. The combination increased iodine uptake in five patients, enabling therapeutic RAI administration in four of them. Notably, two patients achieved a partial response, while two had stable disease at 6 months, suggesting that targeting HER3-mediated resistance could improve redifferentiation efficacy (54). In addition, recent preclinical findings indicate that mutations in the SWI/SNF chromatin remodeling complex (ARID1A, ARID1B, ARID2, or SMARCB1), commonly observed in advanced thyroid cancers, can promote tumor progression and resistance to redifferentiation therapies. In BRAF V600E-mutant mouse models, loss of key SWI/SNF subunits impaired thyroid differentiation gene expression and prevented vemurafenib from restoring iodine uptake, highlighting a potential mechanism underlying radioiodine refractoriness in certain patients (55).

These findings illustrate two evolving therapeutic paradigms for BRAF V600E-mutant thyroid cancers. While vemurafenib alone has demonstrated meaningful tumor control, its toxicity profile and potential for acquired resistance present challenges. Conversely, the redifferentiation approach provides a less toxic and potentially more durable strategy, but patient selection remains crucial, as only a subset of tumors exhibit sufficient iodine reuptake. Future research should focus on refining combination strategies and predictive biomarkers to optimize outcomes for both approaches.

### Dabrafenib

Dabrafenib is a selective BRAF inhibitor that targets the BRAF V600E and V600K mutations in the MAPK signaling pathway, making it effective in a broader range of cancers.

The development of dabrafenib as a targeted therapy for thyroid cancer has transformed the treatment landscape for BRAF-mutant thyroid malignancies, particularly ATC and RAI-refractory DTC. Among the three main MAPK pathway inhibitors studied in this setting – selumetinib, vemurafenib, and dabrafenib – it is dabrafenib, especially in combination with trametinib, that has shown the most transformative clinical impact (Table 6). In several studies, trametinib, a MEK inhibitor, has been added to dabrafenib to enhance efficacy by blocking the MAPK pathway at two levels, reducing resistance, and prolonging tumor control. This combination has proven particularly effective in BRAF V600E-mutant ATC and refractory DTC, where durable responses had previously been elusive.

**Table 6:** Dabrafenib clinical studies summary.

Study	Phase	Population (*n*)	Treatment	Primary endpoint	Key clinical outcomes
Falchook *et al.* (56)	I	BRAF-mutant solid tumors including PTC (*n* = N/A)	Dabrafenib	ORR	50% response rate in BRAF-mutant PTC; median PFS: 5.5 months
Rothenberg *et al.* (63)	II	BRAF V600E-mutant iodine-refractory PTC (*n* = 10)	Dabrafenib + RAI	Restoration of IU	60% restored IU; 2 PRs and 4 SD after RAI
Falchook *et al.* (57)	I	BRAF V600E-mutant metastatic thyroid cancer (*n* = 14)	Dabrafenib	Safety and ORR	29% PR; 64% had ≥10% tumor shrinkage; median PFS: 11.3 months
Subbiah *et al.* (58)	II	BRAF V600E-mutant ATC (*n* = 16)	Dabrafenib + trametinib	ORR	69% ORR; 12-month OS: 80%, PFS: 79%
Subbiah *et al.* (59, 60)	II	BRAF V600E-mutant ATC (*n* = 36) + other rare tumors (ROAR study)	Dabrafenib + trametinib	ORR	56% ORR in ATC; 3 CRs; median OS: 14.5 months; 12-month OS: 51.7%
Busaidy *et al.* (62)	II	BRAF-mutant RAIR-DTC (*n* = 53)	Dabrafenib vs Dabrafenib + trametinib	ORR	42% ORR (dabrafenib); 48% (dabrafenib + trametinib); no PFS advantage detected
Leboulleux *et al.* (64)	II	BRAF V600E-mutant RAIR-DTC (*n* = 24)	Dabrafenib + trametinib + RAI	Restoration of IU + ORR after RAI	95% restored IU; 38% PR after RAI; 12-month PFS: 82%
Toro-Tobon *et al.* (65)	RS	BRAF-mutant RAIR-DTC (*n* = 33, 19 on dabrafenib)	Dabrafenib + trametinib + RAI	Restoration of IU	57.6% restored IU; 20% tumor shrinkage at 6 months post-RAI
Shimoi *et al.* (61)	II	BRAF V600E/R or non-V600 BRAF-mutant solid tumors, including thyroid cancer (*n* = 10 DTC + 5 ATC) (BELIEVE trial)	Dabrafenib + trametinib	ORR	DCR: 84%; median PFS: 6.5 months

RS, retrospective study; N/A, not available; ORR, overall response rate; IU, iodine uptake.

Initial evidence supporting the efficacy of BRAF inhibition in thyroid cancer emerged from early clinical trials in melanoma, where dabrafenib showed high response rates in BRAF-mutant tumors (56). Clinical outcomes specific to thyroid cancer were later reported in a dedicated subanalysis of a phase I trial including 14 patients with BRAF V600E-mutant metastatic thyroid carcinoma, of whom 93% had received prior radioactive iodine (57). The study showed a 29% partial response rate, while 64% experienced at least 10% tumor shrinkage, and median PFS was 11.3 months. Responses were more durable in well-differentiated histologies, and treatment was generally well tolerated, with most adverse events being grade 1.

A major breakthrough came with a phase II trial in BRAF V600E-mutant ATC, a highly aggressive and historically treatment-resistant cancer. Among 16 ATC patients, the study reported an overall response rate (ORR) of 69% (11/16 patients), with a median follow-up of 47 weeks (58). Notably, seven patients had ongoing responses, while the 12-month PFS was 79%, and the 12-month OS was 80%, a significant improvement in a disease where survival was previously measured in weeks. These results led to the FDA approval of dabrafenib plus trametinib in 2018 for BRAF V600E-mutant ATC, marking the first targeted therapy for this highly lethal malignancy. The updated ROAR basket study reinforced these findings in 36 ATC patients, reporting an ORR of 56%, with three complete responses and 17 partial responses. The median OS was 14.5 months, with 12-month and 24-month OS rates of 51.7 and 31.5%, respectively, confirming the long-term survival benefit of this combination (59). These data were subsequently incorporated into a larger basket study that included additional BRAF V600E-mutant rare cancers, with the ATC cohort unchanged, thereby reinforcing the therapy’s central role in thyroid cancer management (60).

In addition, a Japanese cross-organ phase II trial, the BELIEVE trial, including ten DTC and five ATC thyroid cancer patients with BRAF V600E/R or non-V600 BRAF mutations, reported a disease control rate (DCR) of 84% and a median PFS of 6.5 months, reinforcing the broader applicability of dabrafenib–trametinib in BRAF-driven thyroid tumors (61). Interestingly, a phase II randomized trial compared dabrafenib alone vs dabrafenib plus trametinib in 53 patients with BRAF V600E-mutant RAI-refractory DTC, reporting similar ORRs of 42 and 48%, respectively, and no significant difference in PFS (10.2 vs 11.4 months). Despite the proven synergy of this combination in melanoma and lung cancer, the addition of trametinib did not offer a clear clinical advantage in this thyroid cancer setting. The authors suggest that tumor-specific factors, such as lower MAPK pathway dependence or feedback mechanisms, may underlie this modest efficacy. These results imply that dabrafenib monotherapy may be a valid treatment option in RAIR-DTC and highlight the need for better stratification or alternative combination strategies in this population (62).

Finally, in 2022, an important milestone was the FDA’s histology-agnostic Accelerated Approval of dabrafenib plus trametinib for unresectable or metastatic BRAF V600E-mutant solid tumors, largely based on the ROAR basket trial and subsequently strengthened by the BELIEVE trial (59, 61). This decision paves the way for broader applications of this therapeutic strategy in BRAF-mutant thyroid cancers beyond ATC, including papillary and poorly differentiated thyroid carcinomas, which collectively account for a large proportion of BRAF-driven thyroid tumors. It marks a paradigm shift, establishing BRAF-targeted therapy as a cornerstone in the modern management of thyroid cancer.

A second therapeutic approach in refractory DTC using dabrafenib has focused on redifferentiation therapy – restoring RAI avidity through targeted MAPK inhibition. One of the earliest demonstrations came from a phase II pilot study of dabrafenib monotherapy in ten patients with BRAF V600E-mutated iodine-refractory PTC, where 60% of patients showed restored iodine uptake. Following RAI therapy, two patients had partial responses, and four achieved stable disease. In addition, thyroglobulin levels decreased in four of six evaluable patients, indicating biochemical response and resensitization to RAI (63).

These results led to a more advanced phase II study investigating dabrafenib plus trametinib in 24 patients with BRAF V600E-mutant RAI-refractory DTC, which demonstrated restored iodine uptake in 95% of cases. After subsequent RAI therapy, 38% of patients achieved partial responses, with median PFS rates at 12 and 24 months of 82 and 68%, respectively. Notably, ten patients received a second course of treatment, all of whom again regained iodine uptake, suggesting the redifferentiation effect may be transient but repeatable. Importantly, the study addressed whether tumor responses were attributable to the targeted therapy or to the addition of RAI. While significant tumor shrinkage was often observed after 1 month of dabrafenib–trametinib, the authors noted that the durable PFS and lack of correlation between early response and 6-month outcome pointed to the critical role of RAI in achieving lasting tumor control. Thus, the clinical benefit appears to result from the synergistic effect of redifferentiation therapy and subsequent RAI administration (64). Further support for this approach came from a retrospective study at Mayo Clinic, see Table 6 (65).

An important observation in the clinical development of BRAF-targeted therapies is the disparity in antitumor responses between BRAF V600E-mutant ATC and DTC when treated with dabrafenib, alone or in combination with a MEK inhibitor, outside the context of redifferentiation trials. In ATC, treatment with dabrafenib, particularly in combination with trametinib, has led to robust and durable responses, with ORRs of 56–69% and median OS reaching 14.5 months. Conversely, in BRAF V600E-mutant DTC, similar strategies have yielded more modest responses, such as ORRs of 42–48% with no significant PFS advantage, suggesting a less pronounced dependence on MAPK signaling in these tumors. This divergence highlights the necessity to tailor therapeutic strategies not only based on molecular mutations but also considering histological context, tumor aggressiveness, and dependency on oncogenic signaling. Further translational studies exploring MAPK output levels, compensatory pathway activation, and tumor microenvironment differences between ATC and DTC could provide deeper insight into these distinct response patterns.

In summary, the clinical development of dabrafenib, particularly in combination with trametinib, has significantly reshaped the therapeutic landscape for BRAF-mutant thyroid cancers. From restoring RAI sensitivity in DTC to achieving unprecedented survival gains in ATC, this targeted strategy has delivered meaningful clinical benefit across histological subtypes. Importantly, the dual roles of direct antitumor activity and redifferentiation for RAI resensitization are not mutually exclusive but rather complementary strategies, both of which can be pursued sequentially or even simultaneously in the same patient to optimize outcomes. With the support of regulatory approvals, including a histology-agnostic indication, dabrafenib-based therapy now stands as a key pillar in precision oncology for thyroid cancer, offering new hope for patients with previously limited treatment options.

## Concluding remarks

The BRAF V600E mutation has emerged as a central driver in the molecular landscape of thyroid cancer, particularly PTC. Its role in promoting MAPK pathway hyperactivation, tumor dedifferentiation, and impaired iodine metabolism underscores its biological significance. Clinically, BRAF V600E is associated with adverse pathological features and worse clinical outcomes, yet its isolated prognostic value is limited by confounding variables and heterogeneity in tumor behavior. While BRAF V600E can inform clinical decision-making in selected settings – such as solitary intrathyroidal tumors 2–4 cm in size – its utility is significantly enhanced when considered in conjunction with other molecular markers, including TERT promoter and SWI/SNF mutations, or transcriptomic metrics such as the TDS and miRNA profiles.

Looking forward, a more nuanced, multidimensional approach to thyroid cancer management is needed. Integrating genomic, transcriptomic, and epigenetic profiling into clinical workflows will enable more precise risk stratification and personalized treatment strategies. BRAF V600E-targeted therapies, including dabrafenib and trametinib, have already demonstrated substantial benefit in RAI-refractory and anaplastic thyroid cancers, and ongoing trials may extend their indications to earlier disease stages or combination regimens. Moreover, efforts to refine redifferentiation strategies and predict RAI responsiveness using TDS, genetic co-alterations, and miRNA signatures hold promise for improving therapeutic outcomes. Future research should prioritize prospective validation of these integrative models, incorporation into staging systems, and development of cost-effective molecular diagnostics to support widespread clinical adoption. In this evolving landscape, BRAF V600E will remain a cornerstone, but its optimal clinical value will be realized only within a broader framework of precision oncology.

## Declaration of interest

The author declares that there is no conflict of interest that could be perceived as prejudicing the impartiality of this review.

## Funding

The author wants to thank the support of the following grant and institutions: PI23/01986 from the *Instituto de Salud Carlos III, Ministerio de Ciencia, Innovación y Universidades*, Spain, and the Faculty of Medicine, *Universidad Franciso de Vitoria*, Madrid.
